# Phosphorus Leaching in an Acid Tropical Soil “Recapitalized” with Phosphate Rock and Triple Superphosphate

**DOI:** 10.1100/tsw.2010.156

**Published:** 2010-08-03

**Authors:** E. Gikonyo, A.R. Zaharah, M.M. Hanafi, R. Anuar

**Affiliations:** ^1^ Kenya Agricultural Research Institute, Kabete, Nairobi, Kenya; ^2^ Institute of Tropical Agriculture, Universiti Putra Malaysia, Serdang, Selangor, Malaysia; ^3^ Department of Land Management, Universiti Putra Malaysia, Serdang, Selangor, Malaysia

**Keywords:** phosphorus leaching, P recapitalization, massive fertilizer P application

## Abstract

With high rates of phosphorus applied to increase “capital P” as a stock for plant uptake over several years, the question of P leaching is inevitable. We conducted an intact soil column experiment in the field to evaluate P leached from soils treated with triple superphosphate (TSP) and Gafsa phosphate rock (GPR) at 300, 600, and 900 kg P ha^-1^ with and without integration of cattle manure. The lysimeters, made from PVC tubes of 30-cm length, were inserted into the soil up to the 25-cm depth. The tubes were fitted with a resin bag containing a mixture of cation and anion exchange resin (50:50) at the lower end of the tube inserted into the soil. The tubes, arranged in a completely randomized design, were sampled randomly at 10-week intervals for 12 months. Phosphorus extractable from the top- and subsoil at the end of experiment and leached P were determined. More P was leached out from TSP (threefold) compared to GPR, and the amount of P leached increased with increasing rates of P fertilizer applied. Application of manure intensified the amounts of P leached from TSP, particularly at the 6-month sampling time. There was hardly any substantial P leached from the soil treated with GPR. Thus, for effective and efficient long-term P fertilizer management strategies, choosing the right P fertilizer source and monitoring P losses through leaching has to be done for enhanced fertilizer use efficiency and thus reducing P pollution of ground waters.

## INTRODUCTION

One of the strategies proposed for sustainable intensification of food production in the tropics is "phosphorus (P) recapitalization"[1]. This involves massive P application designed to saturate the sorption sites permanently and provide P for crop uptake. This may be achieved through application of a one-time massive dose or by buildup through continuous seasonal small doses. The strategy turns the soil P fixing capacity from a constraint into an asset. Fixed P forms the stock that provides P over a period of 5–10 years referred to as "soil capital", while the P available during an annual cropping cycle is called

"liquid P"[1]. Generally, only ≈10–20% of P applied is available for crop uptake in the year of application ("liquid P"), while the remaining 80–90% of soil P is adsorbed on the soil constituents and builds up as the "soil P capital".

Acid soils in the tropics are dominated by iron and aluminum oxides/hydroxides. Thus, P fertilizer applied to the soil reacts with soil components, mainly hydrous oxides of Fe and Al in the acid soils, forming relatively less soluble compounds. The phosphate ion is negatively charged and, hence, continued P fixation leads to a "semi-permanent" increase in negative charge, resulting in a decrease in the electric potential of the reacting soil particle[2,3]. Consequently, in highly fertilized soils, the bonding strength between soil adsorbents and P decrease because an increasing number of adsorption sites are occupied by the P ions[4]. As a result, increasing P saturation leads to weaker and weaker retention of P, implying that the degree of phosphorus saturation (DPS) appears to govern solution P concentration[5]. The DPS is defined as an index relating P already adsorbed (quantity) by a soil to its adsorption capacity[6].

Generally, if fertilizer P is applied in excess of plant needs, the upper soil layers become saturated with P. Then substantial amounts of P can be leached to deeper soil horizons and/or ground waters. The actual downward movement of P can be described by the "tipping bucket model" as described by Van der Wal et al.[7]. In this model, the soil is visualized as a stack of empty buckets, each bucket representing the sorption capacity of one soil horizon. As P is added to the first layer, the top bucket must be completely filled before any phosphate can flow into the bucket below.

In view of the above, the issue of P loss through leaching with the massive P applied in "P recapitalization" in a single dose or continuous buildup is quite pertinent. However, P has always been considered an immobile P nutrient and, consequently, limited research has been conducted on P leaching. The hypothesis of the work reported in this paper was that when high amounts of phosphate fertilizers (soluble or sparingly soluble) are applied to the soil individually or integrated with organic manure, either or both of the two factors will influence P sorbed and the sorption capacity of the soil and, hence, DPS. Thus, at high DPS levels, there is a possibility of P leaching leading to low fertilizer use efficiency and high costs of production per unit yield. The objectives of this study were to:

1. Determine the magnitude of P leaching in soils treated with two P sources, with and without manure addition
2. Evaluate the relationship between extractable Mehlich-3 P[11] in the soil and P leached

## MATERIALS AND METHODS

### Experimental Site and Materials

The study was conducted at the Puchong Research Farm of the Universiti Putra Malaysia (UPM). The soil is classified as Bungor soil series, a sandy clay loam Ultisol (Typic Kandiudult, clayey, kaolinitic, isohyperthermic) according to Soil Survey Staff (2006)[8]. The experimental area has an average annual precipitation of about 2,200 mm. It is also characterized by a mean monthly maximum and minimum temperature of 32 and 24°C, respectively, and a relative humidity of 70–90%. Topsoil samples air dried and sieved through 2-mm mesh size were characterized by standard methods[9]. The soil and fertilizer characterization is presented in Tables 1 and 2, respectively. The rainfall pattern during the duration of the experiment (July 2003 to July 2004) is presented in Fig. 1.

### Experimental Treatments and Design

Experimental treatments consisted of two P sources: triple superphosphate (TSP) and Gafsa phosphate rock (GPR) at three application rates (300, 600, and 900 kg P $ha^{-1}$) plus control with and without cattle manure at the rate of 20 mg $ha^{-1}$. The experimental design was factorial [(2 fertilizer types × 3 fertilizer

**TABLE 1**
**Characteristics of the Top- and Subsoil Parameter**

| Parameter | Topsoil | Subsoil |
|---|---|---|
| pH water (1:2.5) | 4.5 | 4.5 |
| pH KCl (1:2.5) | 3.9 | 3.9 |
| Total carbon (g $kg^{-1}$) | 30 | 20 |
| Total P (mg P $kg^{-1}$ soil) | 280 | N/D† |
| P sorption maximum (mg P $kg^{-1}$ soil) | 769 | 833 |
| Available P (mg P $kg^{-1}$ soil) by: | | |
| Bray 1 | 4.9 | 2.6 |
| Olsen | 5.2 | 2.6 |
| Pi-strip | 0.6 | 0.4 |
| $Al_2O_3$ (g $kg^{-1}$) | 15.2 | 15.2 |
| $Fe_2O_3$ (g $kg^{-1}$) | 30.8 | 31.6 |
| CEC (cmol (+) $kg^{-1}$) | 5.4 | N/D† |

† Not determined.

**TABLE 2**
**Characteristics of the Fertilizers Used**

| Composition | Fertilizers (g $kg^{-1}$) | | |
|---|---|---|---|
| | GPR | GPR | CIPR |
| P | 204 | 125 | 140 |
| $P_2O_5$ | 462 | 289 | 323 |
| CaO | 192 | 455 | 420 |
| $CaO/P_2O_5$ | 0.4 | 1.57 | 1.3 |
| $Fe_2O_3$ | 6 | 4 | 31 |
| $Al_2O_3$ | 16 | 9 | 107 |
| MgO | 29 | 9 | 23 |
| Solubility in water* | 380 | 0.2 | 0.1 |
| Solubility in 2% citric acid* | 430 | 100 | 87 |
| Solubility in 2% formic acid* | 407 | 110 | 66 |

* Solubility based on total $P_2O_5$.

rates) + 1 control × 2 cattle manure rates] arranged in a randomized complete block with three replications. They were applied in the field in intact soil leaching columns.

The intact leaching columns were constructed using polyvinyl chloride (PVC) tubes of 9-cm internal diameter and 30-cm length (Fig. 2). With the aid of a hammer, the PVC tubes were vertically inserted into the soil to a depth of about 25 cm as shown in Fig. 3a. Care was taken not to disrupt the soil structure. A shovel was then used to remove the soil around the exterior of the tube (Fig. 3b). Next, the soil column was removed by breaking off the core from the main soil body. The PVC containing the soil column was lifted off the pit and the bottom 1.5-cm soil within the PVC tube was removed with a sharp knife and replaced with a resin bag containing cation-anion exchange resin (Fig. 3c). The resin bags were made from a nylon netting fabric (Monyl 58-70 HDX brand, Swiss Silk, Sefar Inc. Printing Division, Switzerland) with a mesh opening of about 0.9 μm. The diameter of the resin bag was approximately equal

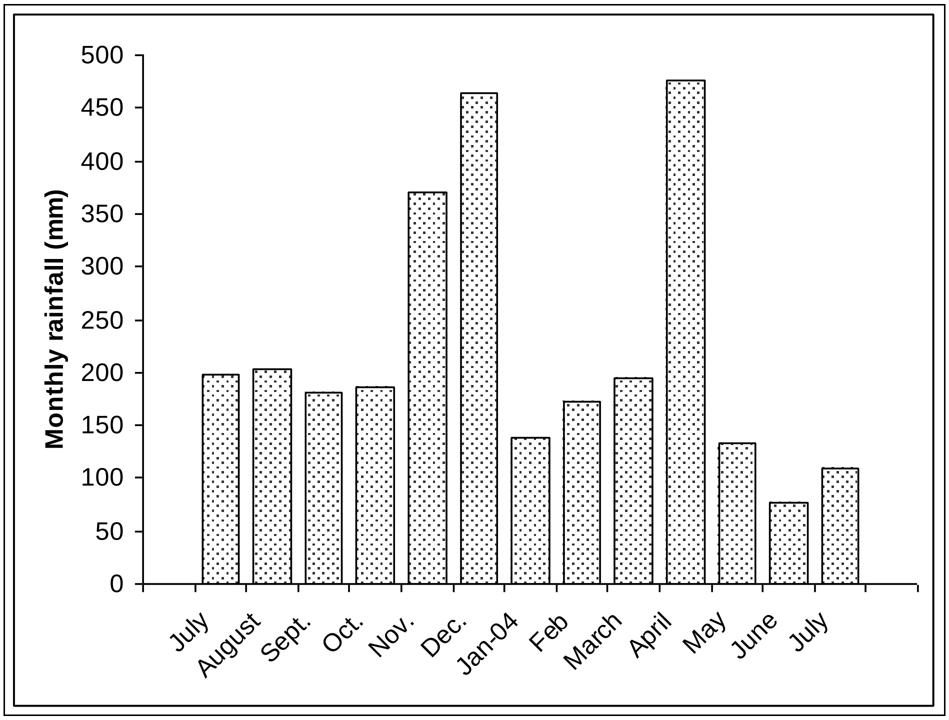

**FIGURE 1.** Rainfall pattern during the experimental period (July 2003 to July 2004).

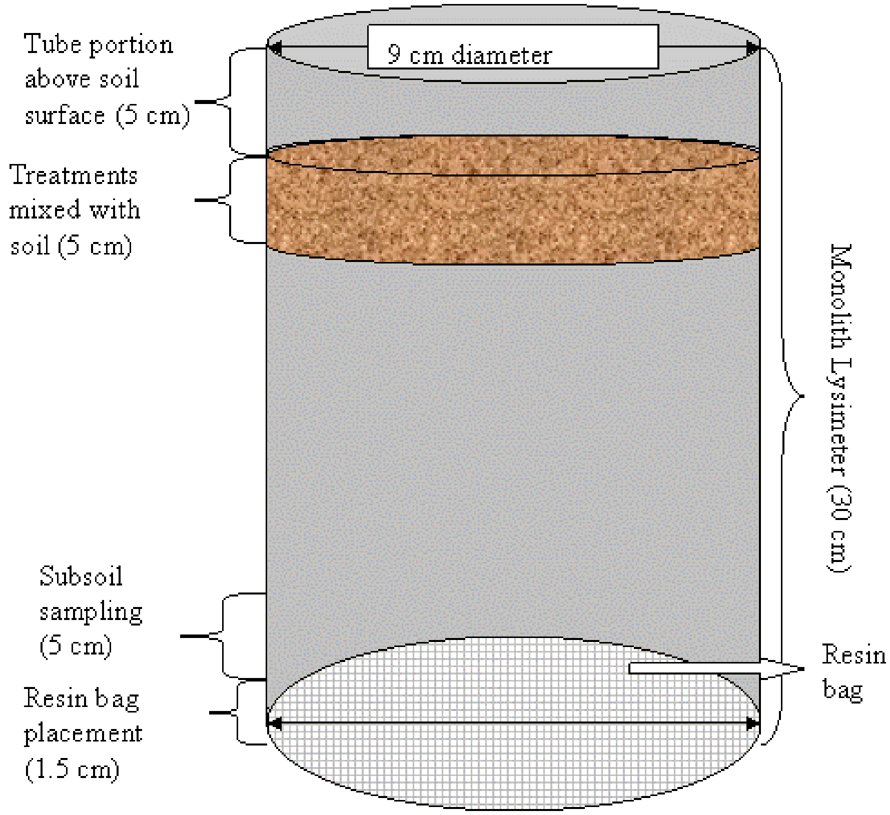

**FIGURE 2.** Schematic diagram showing the intact leaching column modified with ion exchange resin.

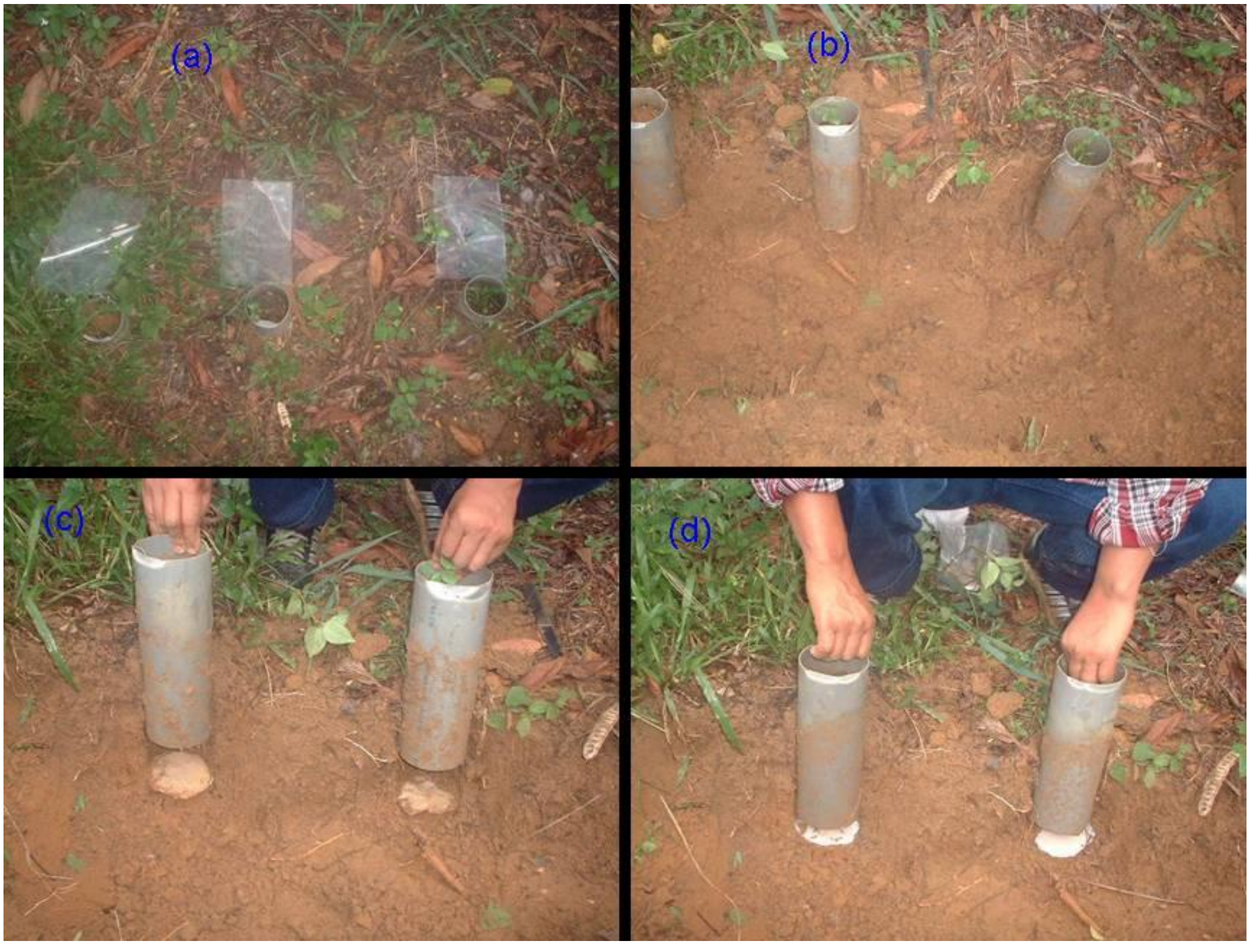

**FIGURE 3.** Sampling procedure showing (a) a view of the PVC tubes as inserted and sampling bags just before sampling, (b) tubes after excavation of surrounding soil showing position of resin bags, (c) old resin bags removed, and (d) replacement of old resin bags with fresh ones.

to the PVC internal diameter (9 cm). They were sealed using an electric plastic sealer, leaving a small perforation through which the cation-anion mixed exchange resin was placed into the bag before finally sealing it.

The cation and anion resins were purchased separately and were hand mixed prior to use. The cation resin (Amberlite IR – 120; Fluka Chemie AG, Buchs, Switzerland) was strongly acid with close-linked polystyrene matrix and $–SO_3H$ as functional group, mesh size 14-52. The cations had an exchange capacity of 1.95 $cmol_c dm^{-3}$. The anion resin (Amberlite IRA-93; Fluka Chemie AG) was a macroreticular, weakly basic resin with tertiary amine groups attached to the cross-linked polystyrene matrix, mesh size 20-50. They had a capacity of 1.40 $cmol_c dm^{-3}$. Cation and anion resins were weighed in equal weights of 12.5 g each, mixed, and placed into the bags. A mixed cation-anion exchange resin was used rather than an anion exchange resin because the former extracts more P than the anion exchange resin alone[10].

One resin bag (25 g) per intact column was then placed flat at the bottom of the soil column covering the whole internal area (Fig. 3d). The whole system of soil column and resin bag was reinserted into the pit and the excavated soil carefully repacked around each PVC tube. The tubes were installed 0.5 × 2 m apart within plots and a total of 42 PVC tubes were installed at the experimental site. Treatments were applied at the top of each soil column and mixed to about 5-cm depth with a knife.

A nondestructive sampling (NDS) method was used with sampling done at 10-week intervals for a total of 50 weeks. At sampling, the PVC tubes were dug out of the soil by removing soil adjacent to the tube (Fig. 3b), and the resin bag resting at the bottom of the tube was removed (Fig. 3c) and placed in a labeled polythene bag. They were replaced with freshly prepared resin bags placed *in situ* until the next sampling time (Fig. 3d). The process was repeated at every sampling. At the end of the experiment, the topsoil (up to 5-cm depth) and subsoil (bottom 5 cm) sampling was carried out.

After removal from the field, the resin bags were air dried and any adhering soil brushed off using a dry brush. Each resin bag was then opened and the cation-anion exchange resins were transferred into a 250-mL centrifuge tube. For P elution, 100 mL of 0.1 M $H_2SO_4$ was added and the mixture shaken on an oscillatory shaker at 200 rpm for 2 h. The suspension was then filtered through filter paper No. 5C and the extract analyzed for P by the ICP (Thermal Elemental IRIS Advantage model). The amount of P leached was calculated using the following formula:

$$\text{P leached (kg P ha}^{-1}\text{yr}^{-1}) = \frac{500[\text{leachate P (mg P L}^{-1}) \times \text{Vol. of extractant (L)}]}{\text{Cross sectional area of tube (cm}^{2})}$$

The air-dried soil samples taken at the end of the experiment were analyzed for P using Mehlich-3 extractant (0.2 M $CH_3COOH$ + 0.25 M $NH_4NO_3$ + 0.015 M $NH_4F$ + 0.013 M $HNO_3$ + 0.001 M EDTA) at 1:20, soil/solution ratio with 5 min of shaking[11]. After filtration, the supernatant was read on the ICP for P.

Analyses of treatment effects on P leached over time were evaluated by combined analysis using the mixed model procedure.

## RESULTS

### Effects of Different Treatments on P Leached

The P leached was significantly influenced by P source ($p$ = 0.0001) and P rate ($p$ = 0.0051), but not influenced significantly by manure addition ($p$ = 0.06). The interactions P source × P rate × manure × time significantly influenced the P leached ($p$ = 0.0005). Phosphorus leached ranged from 0.01 (control) to 3.88 kg P ha$^{-1}$ year$^{-1}$ (TSP-manure combination). More P was leached from TSP than GPR treatments to the tune of threefold P from TSP relative to GPR treatments. In addition, the P amounts leached increased with increasing P application rates (Fig. 4a–d). For example, application of TSP at 300, 600, and 900 kg P ha$^{-1}$ led to 61, 158, and 289% P leaching, respectively, in treated soils relative to control. Sole manure did not increase P leached significantly (Fig. 4a).

Over time, the total amount of P leached was dependent on an interaction of P source × P rate × manure × time (Fig. 4). When manure was integrated with TSP, the magnitude of P leached was not significant at the initial sampling period (10 weeks), but became significant at the 20-week sampling at all the P rates (Fig. 4b–d). At the 10-week sampling, more P was leached from TSP alone than the combination of manure and TSP, while the trend reversed in the next sampling (20 weeks). After 20 weeks, P leached declined at a high rate with an exception of the 600 kg P ha$^{-1}$ rate for the manure and TSP combination, which gave relatively high P leached throughout the experimental period (Fig. 4c). Phosphorus leached in TSP alone was reasonably high at the 600 and 900 kg P ha$^{-1}$ rate at the first 10-week sampling, but declined rapidly to low levels at 20 weeks (Fig. 4c,d). On the other hand, the 20-week sampling coincided with the highest P leached in the TSP-manure combination. No significant P was leached from the GPR-treated soils with an exception of the 600 kg P ha$^{-1}$ rate, where notably low amounts of leached P were recorded at the 20-week sampling (Fig. 4c).

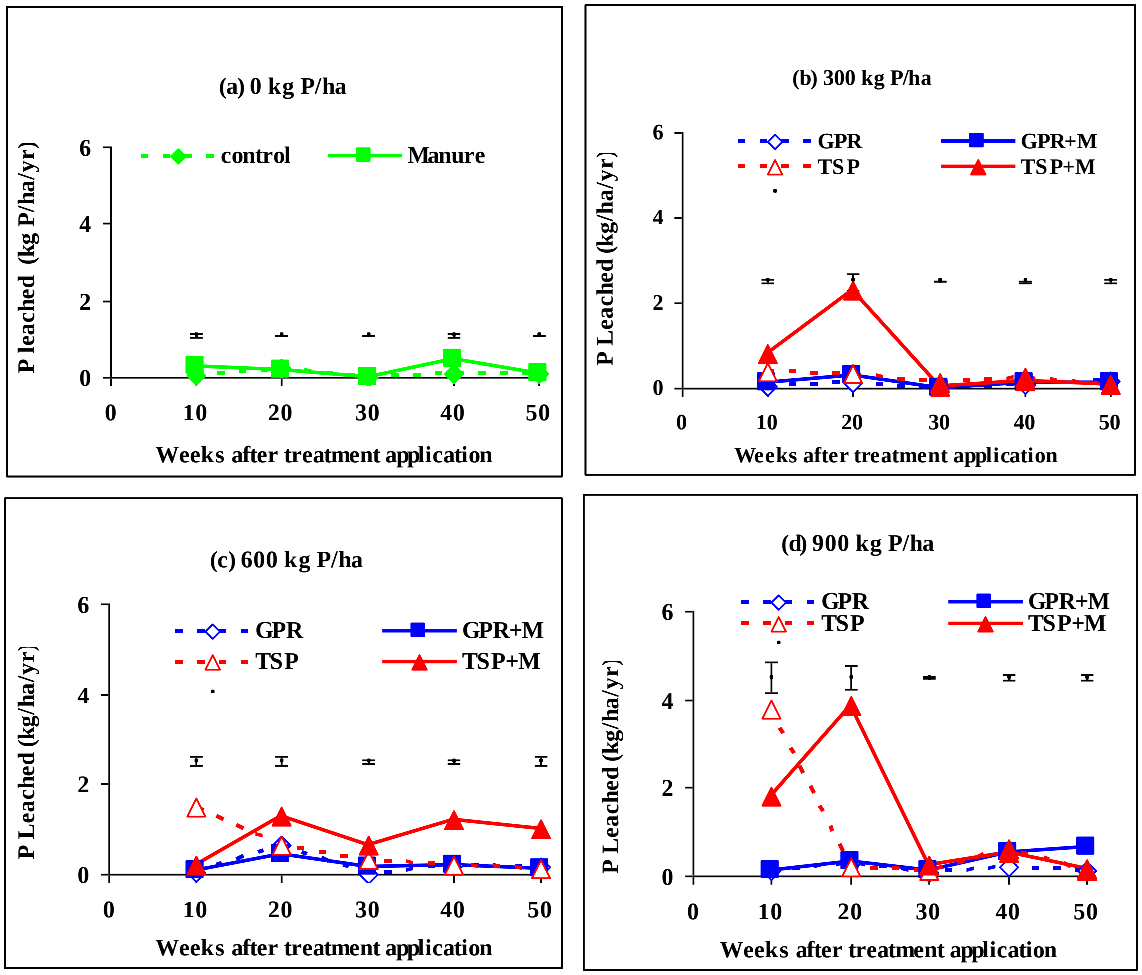

**FIGURE 4.** Effect of P source, plus/minus manure at different P rates on P leached over time. (a) At 0 kg P $ha^{-1}$ (control +/– manure), (b) 300 kg P $ha^{-1}$, (c) 600 kg P $ha^{-1}$, and (d) 900 kg P $ha^{-1}$. The bars denote standard error (S.E).

## Mehlich-3 Extractable P and Ca from the Top- and Subsoil Samples at the End of the Experiment

Mehlich-3 P levels in the topsoil were significantly influenced by P source, P rate ($p = 0.0001$), and their interactions ($p = 0.0005$). However, effect of manure and P source × manure were not significant ($p > 0.05$). TSP-treated topsoil gave an average of 176 mg P $kg^{-1}$ (>200%) compared to 78 mg P $kg^{-1}$ in GPR. Extractable P from the topsoil was related to P application rates in a quadratic relationship in GPR and linear relationship in TSP (Fig. 5a). The linear relationship between extractable P in TSP and TSP-manure combination–treated soils were described by the equations $y = 41.16 + 0.33x$ and $y = 38.7 + 0.27x$, respectively. It is important to note that sole TSP-treated soils exhibited higher extractable amounts of P than TSP + manure (Fig. 5a). Alternatively, in GPR, the quadratic relationship exhibited by extractable P showed increased amounts of Mehlich-3 P from a minimum (≈ 20 mg P $kg^{-1}$) in the control to a maximum (147 mg P $kg^{-1}$) at 600 kg P $ha^{-1}$ + manure, and then declined at 900 kg P $ha^{-1}$. Equations describing Mehlich-3 P in GPR and GPR-manure combinations were $y = 15.26 + 0.25x – 0.00002x^2$ and $y =24.37 + 0.51x – 0.0005x^2$, respectively. It is remarkable that contrary to the observation in TSP + manure and sole TSP, GPR + manure exhibited higher extractable P levels than sole GPR (Fig.5a).

Similar to the topsoil, extractable Mehlich-3 P in the subsoil was significantly influenced by the application rate of fertilizer ($p = 0.017$), but unlike the topsoil, manure addition significantly influenced Mehlich-3 P ($p = 0.031$) and the effect of P source was not significant ($p = 0.15$) in the subsoil. Nevertheless, the subsoil P levels were a function of P source × P rate × manure interaction ($p = 0.017$).

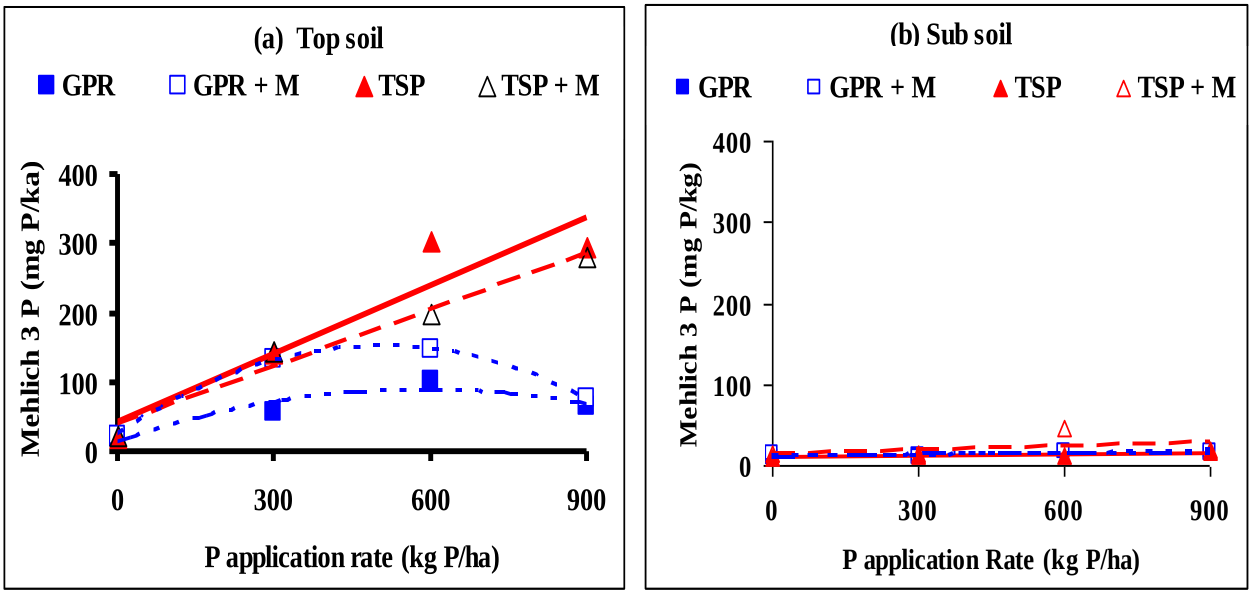

**FIGURE 5.** Relationship between Mehlich-3 extractable P and different rates of P applied in the (a) top- and (b) subsoil.

The subsoil P levels varied closely from 10.7 (control) to 45.7 mg P $kg^{-1}$ (TSP, 600 kg P $ha^{-1}$ + manure). On average, addition of manure increased subsoil P from 13.33 to 18.7 mg P $kg^{-1}$ (≈40%), while fertilizer addition increased extractable soil P from 12.3 (control) to 22.8 mg P $kg^{-1}$ (600 kg P $ha^{-1}$) as shown in Fig. 5b. Nevertheless, only P increase at 600 kg P $ha^{-1}$ was statistically different from control.

The results showed a distinct difference between the TSP-manure combination and other treatments (Fig. 5b). The P levels increased linearly ($y = 15.3 + 0.016x$) with increasing P rate, while the increase in other treatments were not significant. This implies that the highest P leaching occurred in the TSP + manure treatment.

Soil Ca levels in the topsoil, unlike P, were only influenced significantly by P source ($p$ = 0.048), while the effect of fertilizer rate was not significant ($p$ = 0.073). Soil Ca levels ranged between 428 (control) and 928 mg Ca $kg^{-1}$ (GPR at 600 kg P $ha^{-1}$ + manure). Higher average soil Ca levels were extracted from GPR-treated soils (703 mg Ca $kg^{-1}$) than TSP-treated soils (541 mg Ca $kg^{-1}$) by about 30%. Manure alone did not increase the soil Ca levels significantly. In the subsoil, Ca was only influenced significantly by the rate of fertilizer application. Similar to P, Ca increased with increasing fertilizer rate from 143 (control) to 258 mg Ca $kg^{-1}$ (80%) at 600 kg P $ha^{-1}$, coinciding with the maximum Ca level in the topsoil. Similar to the topsoil, Ca declined slightly at 900 kg P $ha^{-1}$ (data not shown).

## Correlation between Various Parameters

Mehlich-3 P extracted from the topsoil was significantly correlated to the P levels in the subsoil (r = 0.95, $p$ = 0.0001) as shown in Fig. 6a. In addition, it was also correlated to the average P leached (r = 0.86, $p$ = 0.0001) as shown in Fig. 6b. Both subsurface soil P and average P leached increased with increasing topsoil extractable P in a linear fashion as shown by the equations in Fig. 6a and 6b, respectively.

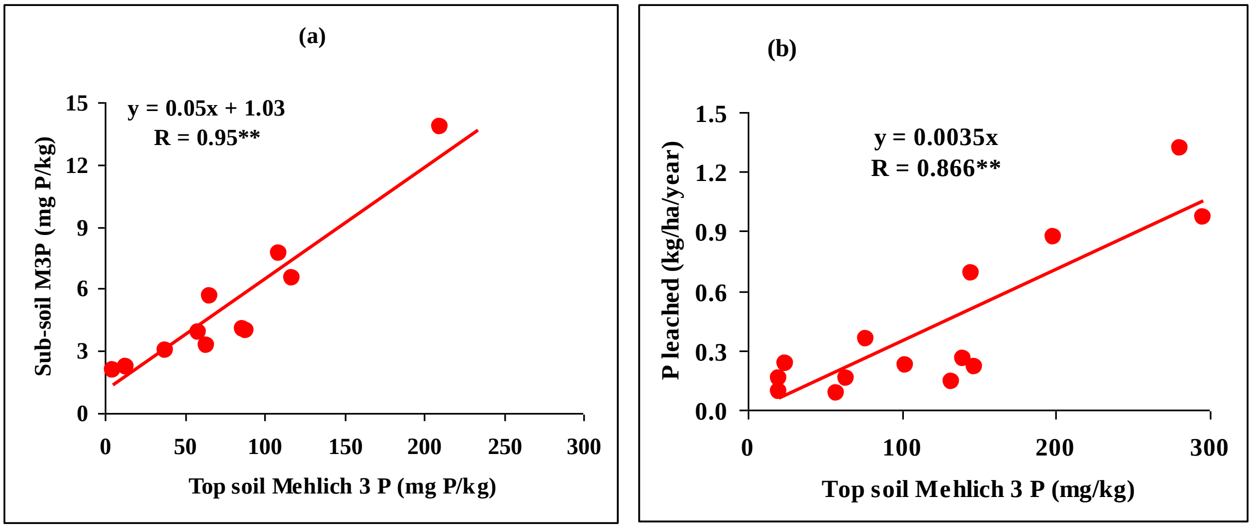

**FIGURE 6.** Relationship between Mehlich-3 P extractable from the topsoil vis-a-vis (a) subsoil Mehlich-3 P and (b) total P leached.

## DISCUSSION

### Phosphorus Leached in Relation to P Sources, P Rates, and Manure Application in the Leaching Columns

More P was leached from the TSP treatments than GPR treatments owing to the higher solubility of TSP relative to GPR (Table 2). The TSP was 2000-fold as soluble as GPR in water, while it was fourfold as soluble in both 2% citric and 2% formic acids. In the absence of manure, P leached in TSP increased with increasing P rates and declined rapidly, particularly at 900 kg P $ha^{-1}$. The highly soluble TSP may have led to higher P saturation of the soil sorption complex as evidenced by the higher Mehlich-3 P levels in the topsoil compared to GPR (Fig. 5a).

From previous findings, increasing P saturation is associated with decreasing bonding strength between soil adsorbents and P, resulting in the continued weakening of P retention and hence high P leached[4]. The high and rapid decline of P leached at the 900 kg P $ha^{-1}$ rate could probably be ascribed to very high P saturation, most likely above the critical threshold point[12]. For GPR, P leaching was only recorded at the 600 kg P $ha^{-1}$, but not at the 300 or 900 kg P $ha^{-1}$ rates, likely due to very low available P at the lower rate of 300 kg P $ha^{-1}$ and also low available P at 900 kg P $ha^{-1}$ despite the high rate of application due to lower GPR solubility at the higher application rate. Apparently, the quadratic relationship between extractable Mehlich-3 P and P application rate is evident of the decreasing GPR dissolution with P rate.

A combination of manure with GPR resulted in an insignificant P leaching despite the P increase in the topsoil Mehlich-3 P (Fig. 4). Nevertheless, the magnitudes of P levels were still lower than those of TSP and, consequently, the P saturation was still lower than TSP; hence, no significant P leached. Alternatively, manure addition to TSP led to distinctly higher P leached, particularly at the 6-month sampling, but declined at a fast rate to lower levels at the 9-month sampling time (Fig. 4). The low P leached at the first sampling (3 months) was likely due to P immobilization by the micro-organisms associated with increased organic matter. The increase afterwards could be explained by additional P from manure mineralization, reduced P fixation of the soil[13], all resulting in higher P saturation coupled with the fact that organic P has higher mobility relative to inorganic P[13]. Similar findings of higher P leaching in treatments with manure than without were also reported in the literature[14].

### Phosphorus Leached Based on Extractable Mehlich-3 P in the Top- and Subsoil Samples

The topsoil P levels in TSP treatments increased linearly with increasing P rates, but the TSP-manure combination exhibited lower P than the sole TSP treatment. Alternatively, in the subsoil, Mehlich-3 P was higher in the TSP + manure treatments than the sole TSP treatment, and the two were related as shown by the correlation coefficient (R = 0.95**). This most likely is an indication of higher P leaching from the topsoil, subsequently accumulating in the subsoil (Fig. 6a). Thus, increase in subsoil P must have been the result of a downward movement of fertilizer P. This was further affirmed by the relationship between P in the topsoil and P leached (R = 0.87**). Comparable results of fertilizer P leaching downwards from the topsoil to the subsoil have been previously reported[15,16]. Highest P losses above soil Mehlich-3 P concentrations of more than 100–130 mg P $kg^{-1}$ was also reported[17]. In our experiment, Mehlich-3 P was above 100 mg P $kg^{-1}$ in TSP, but below for GPR, which explains why P loss was higher in the TSP- than GPR-treated soils.

In sole TSP-treated soils, there was no difference in Mehlich-3 P in the subsoil and control treatment due to lower amounts of P leached down the soil profile, probably due to high fixation of P by the soil, in the absence of manure, thus limiting the P leached. Some P leaching was observed from the sole TSP treatments for the first 20 weeks in the columns. The inconsistency may be explained in the literature[18]. The study reported one situation of P leaching that is dominated by preferential flow transport P, i.e., water transport occurring rapidly through cracks, wall flow, and biopores; hence, only a small part of the subsoil interacts with the P. Thus, the leachate bypasses most of the sorbing area of subsoil. In our experiment, preferential flow may have been dominant as deduced from the high coefficients of variation ranging between 84 and 176% for the different sampling times; thus, resulting in significant P leached without P accumulating in the subsoil. However, in TSP + manure integration, the higher amount of P due to additional P from manure mineralization, coupled with reduced P fixation, some P may still have been lost through the matrix and hence the P accumulation observed. Similar to the current findings, Bolland et al.[19] reported highest P levels in soils treated with superphosphates (SSP) and lowest for PR, and they also observed P leaching downward in the soil profile.

## CONCLUSIONS

It is evident that P can be leached when applied at very high rates, particularly the soluble TSP, and leaching is much higher when TSP is integrated with manure. However, although farmers do not apply such high P levels as used in this experiment, repeated P applications may result in the progressive saturation of adsorption sites; hence, leading to P leaching. No significant leaching occurred in GPR, implying that it would be environmentally safe to use PRs even at high application rates or repeatedly over long periods of time.

Levels of Mehlich-3 P in the top- and subsoil could be used to predict the P leaching potential of the soil. However, the profile soil characteristics may fail to reveal the P leaching potential, where P is dominantly water translocated through preferential flow pathways. In such cases, leaching columns may be preferred or could complement the soil profile P leaching assessment.

Consequently, effective and efficient long-term P management strategies are needed to monitor P leaching for enhanced fertilizer use efficiency and reduced environmental pollution, particularly of ground water.

## ACKNOWLEDGMENTS

We are grateful to the Institute of Tropical Agriculture for offering a postdoctoral fellowship and to the Third World Organization of Women in Sciences (TWOWS) for the Ph.D. fellowship offered to the first author, Esther W. Gikonyo. The research was conducted at the Department of Land Management, Faculty of Agriculture, Universiti Putra Malaysia as part of a doctorate degree.
